# Hybrid structure of white layer in high carbon steel – Formation mechanism and its properties

**DOI:** 10.1038/s41598-017-13749-7

**Published:** 2017-10-16

**Authors:** Rumana Hossain, Farshid Pahlevani, Evelien Witteveen, Amborish Banerjee, Bill Joe, B. Gangadhara Prusty, Rian Dippenaar, Veena Sahajwalla

**Affiliations:** 1 Centre for Sustainable Materials Research and Technology, School of Materials Science and Engineering, UNSW, Sydney, Australia; 2 University of Delft and visiting researcher, School of Mechanical, Materials, Mechatronic and Biomedical Engineering, Faculty of Engineering & Information Science, University of Wollongong, Wollongong, Australia; 3 School of Mechanical and Manufacturing Engineering, UNSW, Sydney, Australia; 4 School of Materials Science and Engineering, UNSW, Sydney, Australia; 5 School of Mechanical, Materials, Mechatronic and Biomedical Engineering, Faculty of Engineering & Information Science, University of Wollongong, Wollongong, Australia

## Abstract

This study identifies for the first time, the hybrid structure of the white layer in high carbon steel and describes its formation mechanism and properties. The so-called ‘white layer’ in steel forms during high strain rate deformation and appears featureless under optical microscopy. While many researchers have investigated the formation of the white layer, there has been no definitive study, nor is there sufficient evidence to fully explain the formation, structure and properties of the layer. In this study, the formation, morphology and mechanical properties of the white layer was determined following impact testing, using a combination of optical and SE- microscopy, HR-EBSD, TKD and TEM as well as nano-indentation hardness measurements and FE modelling. The phase transformation and recrystallization within and near the white layer was also investigated. The microstructure of the steel in the white layer consisted of nano-sized grains of martensite. A very thin layer of austenite with nano sized grains was identified within the white layer by HR-EBSD techniques, the presence of which is attributed to a thermally-induced reverse phase transformation. Overall, the combination of phase transformations, strain hardening and grain refinement led to a hybrid structure and an increase in hardness of the white layer.

## Introduction

Steel that has been subjected to high strain rates and/or impact deformation often displays a thin, so-called ‘white layer’ that appears featureless in optical microscopy. This white layer is hard and brittle; it increases the steel’s propensity towards spalling or chipping, thereby limiting industrial applications. While many researchers have investigated the formation of this ‘white layer’, there has been neither definitive study, nor experimentally validated evidence that fully explains the formation, structure and properties of this ‘white layer’.

It seems that the high strain rate deformation causes significant changes to the structure of the outermost layer of such steels, creating an etch-resistant layer which appears white and flawless, with no grain boundaries visible under an optical microscope. Numerous studies have focused on the white layer in different manufacturing applications such as hard turning^1,2^, drilling^3^, reaming^4^, grinding^2^, electric discharge machining and various characteristics of this layer have been described. Being harder than martensite^5^, the white layer is generally considered a detrimental phase for many engineering applications because of its low ductility, extreme hardness and its vulnerability to chipping^6^. However, no study has to date provided comprehensive understanding of the formation mechanism, the mechanical characteristics and the crystal structure of the white layer and hence, there is an urgent need to resolve these uncertainties^5,7–10^. Moreover, it does not seem that any previous study has specifically addressed micro- to nano-scale understanding of the mechanisms of formation of the white layer in high carbon steel under conditions of direct impact and attrition. Earlier research on high carbon martensitic steel that was subjected to impact loading has shown that textural changes may occur and that the mechanical properties in the vicinity of the white layer are modified^8^.

The structure of the white layer and adjacent area in high carbon steel seems to consist of different layers of untempered martensite, over-tempered martensite and bulk material^11^. Furthermore, the white layer is harder than the dark layer, which separates the white layer and the unchanged bulk material,^12^. The existence of a softer dark layer has been attributed to a thermally-induced reverse phase transformation of retained austenite^13^. The hardness of the white layer is attributed to significant grain refinement up to the nano-level^14^. However, such large scale ultra-fine grain formation might lead to deterioration in ductility, potentially leading to spalling and consequently, metal loss.

From an industrial point of view, the present study was aimed at evaluating low cost, low alloyed, high carbon steel as a wear resistant material for use under extreme impact operating conditions. In order to assess, the microstructural response of these steels to severe plastic deformation down to the nano-metre level, we used a variety of characterization techniques including optical microscopy, secondary electron microscope (SEM), X-ray diffraction (XRD), high resolution electron backscattering diffraction (HR EBSD), transmission Kikuchi diffraction (TKD), transmission electron microscopy (TEM) and focused ion beam (FIB) sectioning.

## Experimental Procedure

**Figure 1:**
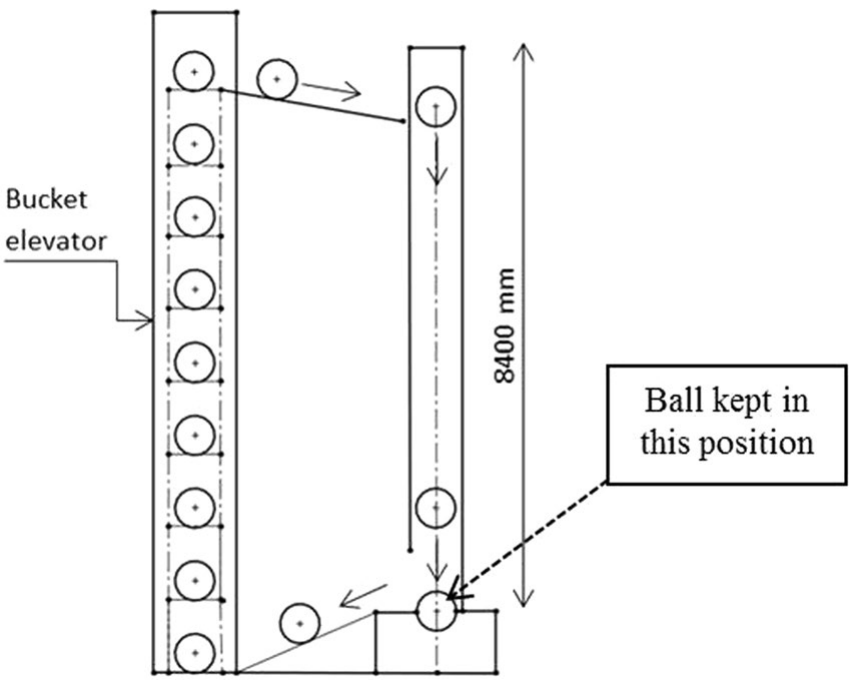
Schematic diagram of the impact test.

A commercial grade, high-carbon steel (1.0%C) was investigated, which in the as-received condition exhibits a mixture of plate and lath martensite and containing 30–40% retained austenite. The high strain rates required for the formation of a white layer was achieved at room temperature in a locally developed drop-ball test, schematically shown in Fig. 1. Steel balls, 125mm diameter, were lifted by a bucket elevator and then allowed to fall freely from a height of 8400 mm onto another ball, kept on the bottom of the tool die. This cycle was repeated several hundred times in a single experiment.

Standard metallographic wet grinding and polishing methods were used to prepare the samples for optical microscopy. A PANalytical Empyrean XRD instrument was used with unfiltered Co-Kα radiation at 45 kV and 40 mA current for quantitative XRD to measure the volume fraction of phases from a 2θ spectrum that was acquired at a step size of 0.0260 over an angular range of 45° to 110°. Samples for scanning electron microscopy were coated with a thin layer of platinum, which enabled observation of the edges of samples without drift in the SEM. In addition to the observation of the very edges of the samples, crack formation could be identified. Since a single SEM-image of the edge of a sample cannot provide sufficient information, a series of pictures were taken of a specific edge. These pictures were then cropped and merged using Photoshop CS6 software. High resolution backscattered electron based orientation microscopy and transmission Kikuchi diffraction (TKD) investigations of the stress induced samples were conducted using an Oxford system with an attached Carl Zeiss AURIGA® CrossBeam® field emission gun scanning electron microscopy (FEG SEM) workstation. Microstructures of the deformed samples were also observed using a TEM equipped with a field emission gun (Philips CM 200, Netherlands). All the TEM and TKD samples were prepared using a dual beam FIB (FEI xT Nova Nanolab 200, USA). The thickness of the specimens was estimated to be around 70nm – 100 nm.

Orientation Imaging Microscopy (OIM) analysis version 8 has been used for dislocation mapping and Orientation Imaging Microscopy (OIM) analysis version 5.2 and 7 has been used for KAM maps.

The EBSD scan was conducted with a pattern binding of 2 × 2, with an integration number of frames of 10 for 20 kV and the step size chosen was 100 nm and for TKD analysis, step size was chosen 5 nm. A clean-up procedure for OIM (EBSP) micrographs has been done by Dilation clean up method. To perform clean-up on this data we used a single step process setting 5 degrees for the Grain Tolerance angle (the default), 5 for the Minimum Grain Size. The hit rate for the EBSD was more than 80% and the fraction of replaced points for images was 2–7%. The average CI values greater than the tolerance value (0.1 in this case) were confirmed.

### Numerical Simulation

It is well known that white layer formation is the result of localized high impact stresses^15^, but it has always been a challenge to predict the numerical values of the stresses generated. To this end, a non-linear finite element (FE) model was developed in ANSYS 16.2 using explicit dynamics applying the multilinear isotropic hardening plasticity rule. The main aim of developing the model was to predict the optimised range of the stresses generated during the formation of a white layer. The input parameters (plastic strain and the corresponding stress) for this FE model were obtained by conducting a compression test at room temperature at a quasi-static strain rate of 10^−4^/s. Post-processing of the model was carried out to predict the von-Mises stresses occurring at the impacted surface of the grinding balls. To the authors knowledge, analytical equations for elastic-plastic flow behaviour of material are not well defined and hence, the model was validated using a convergence technique in order to determine the optimum stresses generated during impact. These stresses were then compared to the published literature, which estimated the stresses required to form a white layer.

For a realistic representation of the arrangement of the impact test in ANSYS, the balls were kept apart at a distance of 87.5mm. The force acting on the ball during its free fall and the velocity of the ball at that particular moment were determined using equations 1 and 2 respectively. The bottom ball was fixed in order to restrict its movement. The corresponding value of force and velocity were calculated as:

1 $$F=mg=78.71\,N$$

2 $$v=\,\sqrt{2gh}=\sqrt{2\ast 9.81\ast (8.4-87.5)}=12.78\,m/s$$

**Figure 2:**
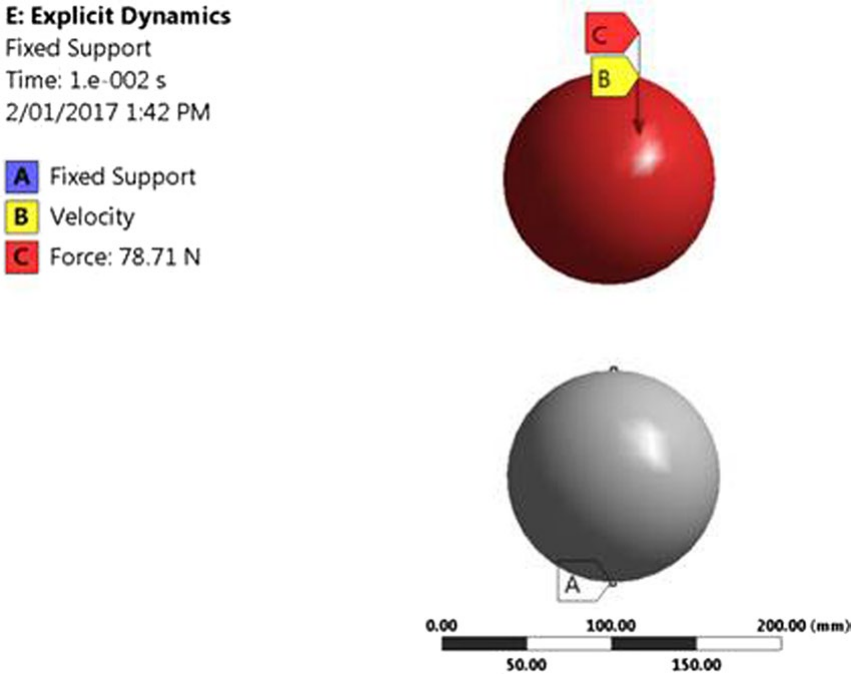
Boundary conditions applied to the free fall grinding balls.

Meshing of the model was done to define its efficiency. As mentioned earlier, in order to predict the optimised values of stress generated due to the impact deformation, successive iterations for the developed model was done by changing the elemental size. Triangular element was chosen for the meshing of the geometry and the transition ratio for the inflation of the mesh was kept at 0.272. Figure 2 shows the arrangement of the impact test and the boundary conditions applied.

## Results and Discussion

### Base Material

**Figure 3:**
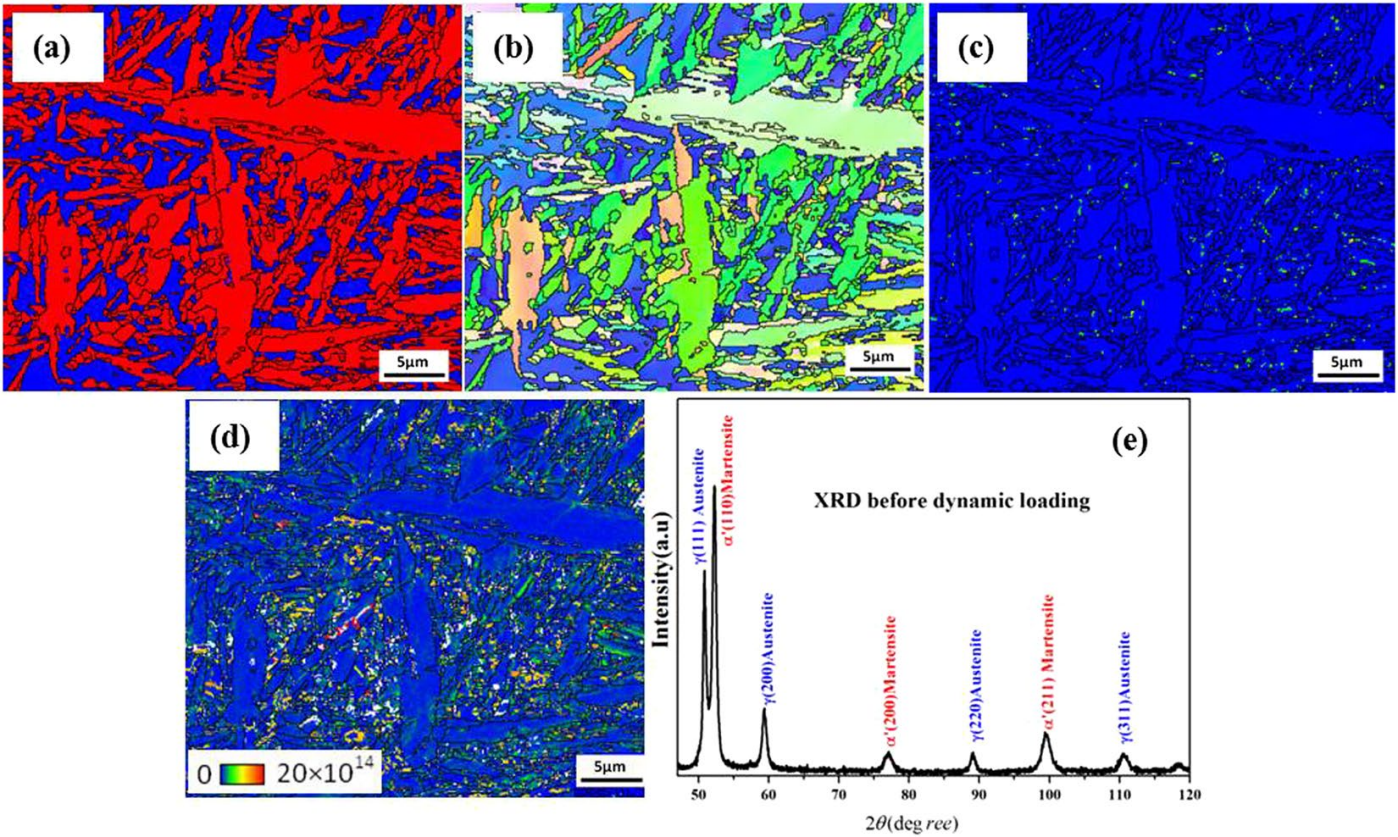
EBSD micrograph and XRD spectrum of the material before impact test. (**a**) EBSD micrograph, here red is martensite and blue is retained austenite (**b**) Inverse pole figure (IPF) mapping (**c**) KAM map and (**d**) dislocation mapping. Black lines are grain boundaries with a misorientation angle of more than 15°. (**d**) XRD spectrum of the base material.

Figure 3 shows the EBSD micrograph of the undeformed sample. In the phase map, martensite is shown in red and the retained austenite in blue. The black lines on the maps represent the boundaries, across which misorientation is more than 15º. Overall, the EBSD micrograph of the undeformed sample revealed the presence of retained austenite with lath and plate-shaped martensite. Prior to the impact test, the specimen contained a substantial fraction (30-40%) of retained austenite. Two types of retained austenite – blocky and film morphologies – were identified and shown in the EBSD micrograph. The inverse pole figure (IPF) mapping and X-ray diffractogram of the specimen before compression indicate uniform texture in the austenite and martensite grains.

The kernel average misorientation (KAM) of the undeformed steel sample was negligible, hence the low dislocation density shown in Fig. 3d. The misorientation close to some of the austenite-martensite grain boundaries shows a shift in the distribution towards higher local misorientations, suggesting that much energy is stored in the vicinity of the austenite/martensite grains boundaries thereby increasing the likelihood of recrystallization nuclei at these positions. Before compression testing, the sample showed large austenitic peaks at (111)γ, (200)γ and (220)γ diffraction positions in the XRD pattern.

The relevant literature shows that when the retained austenite attains sufficient energy from induced compression, randomly spaced overlapping stacking faults create martensite^16^. The α′-martensite phase nucleates at the intersections of shear bands, i.e. dislocation pile-ups on closely spaced slip planes^17–20^. As a result of this transformation, the amount of retained austenite decreases^9,16,21^.

### SEM observation of the White layer

**Figure 4:**
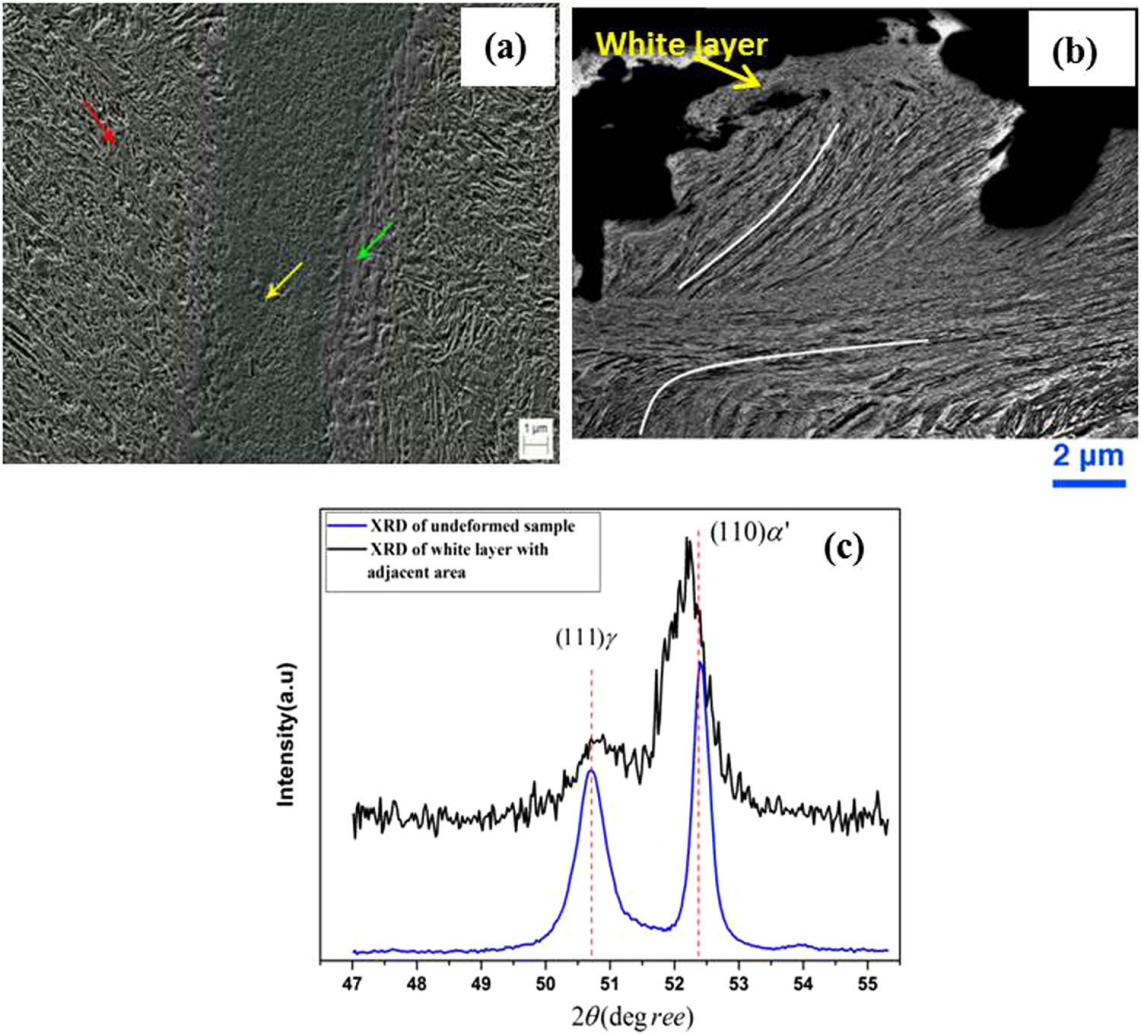
SEM images of the deformed sample featuring the elongated martensite structure and the white layer in (**a**) and (**b**); XRD diffraction pattern for the white layer and adjacent region in (**c**). XRD spectra of the White layer and adjacent region.

Examination of the segmented white layer morphology shows different characteristics at different sites on the sample (Fig. 4a,b). In Fig. 4a the formation of a very fine martensitic structure is clearly noticeable and in Fig. 4b a highly deformed martensitic structure indicates high plastic deformation at the micro-scale.

Figure 4a has been highlighted with different coloured arrows to show the different morphologies inside and outside the white layer. Inside the white layer, marked with the yellow arrow, the nano-sized equiaxed grains are clearly visible. Following deformation, base/bulk material adjacent to the white layer appears to be stress induced martensite as indicated by the red arrow (Fig. 4a). The green arrow marks the region between the white layer and the base material where the morphology is slightly different from both the stress induced martensite of the base material and the ultra-refined grains within the white layer. Figure 4b reveals distinctive flow patterns within the deformed microstructure, and provides convincing evidence that severe plastic deformation occurred, which formed very dense martensite grains. The flow pattern at the top of the figure shows the transition from plastically deformed grains to a refined microstructure, characterised by round, ultra-fine grains marked as ‘white layer’ in Fig. 4b. It is evident that this outer layer can easily be chipped off.

Figure 4c shows the XRD spectra of the white layer and adjacent region following impact testing. The XRD patterns correspond closely to the transformation of austenite to martensite as a result of severe plastic deformation. After deformation, there are variations in the peak intensities and their respective positions due to phase transformations and changes in the sizes of the crystals. Due to the severe plastic deformation, a deformation-induced phase transformation occurred as evidenced by a more pronounced and displaced BCT α’-martensite peak at the (110)α’ diffraction position and a reduced γ-austenite peak at the (111)γ diffraction position. These observations may be attributed to incomplete transformation of retained austenite to martensite or alternatively to newly nucleated austenite grains that formed as a result of the heat generated by impact.

Finite Element modelling

**Figure 5:**
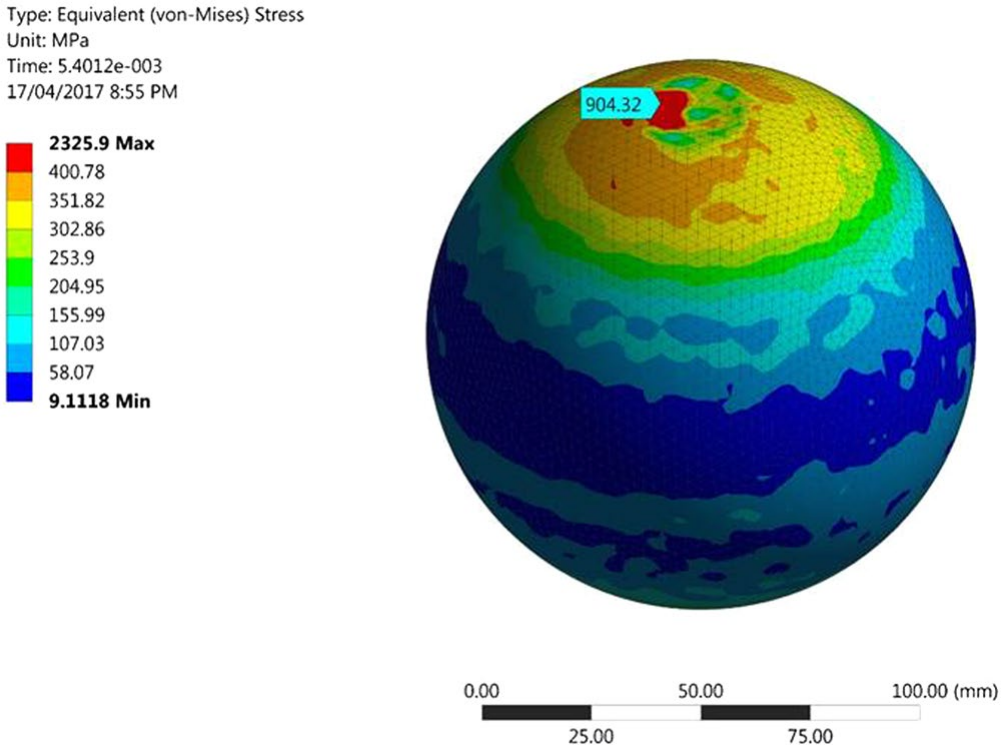
Von-Mises stress at the collision surface.

Non-linear FE analysis was carried out in order to predict the von Mises stresses generated in the ball after impact as a result of impact and the predictions are shown in Fig. 5. The maximum stress generated at the top surface where the collision between the balls occurred was 904.32 MPa when the assigned element size was 3 mm. The fringe range was manually adjusted for better visualisation. Due to the accumulation of this localized stress, the material undergoes severe plastic deformation in a localized zone. As a result of severe plastic deformation and the dissipation of much thermal energy during impact, the white layer is formed. Stress optimization was done for different element sizes 3, 4 and 5 mm respectively, which yielded maximum equivalent stresses of 904.32, 947.25 and 1004.6 MPa respectively. The predicted values are in good agreement with the values predicted by earlier studies^20,21^.

### Crack propagation through the White layer

**Figure 6:**
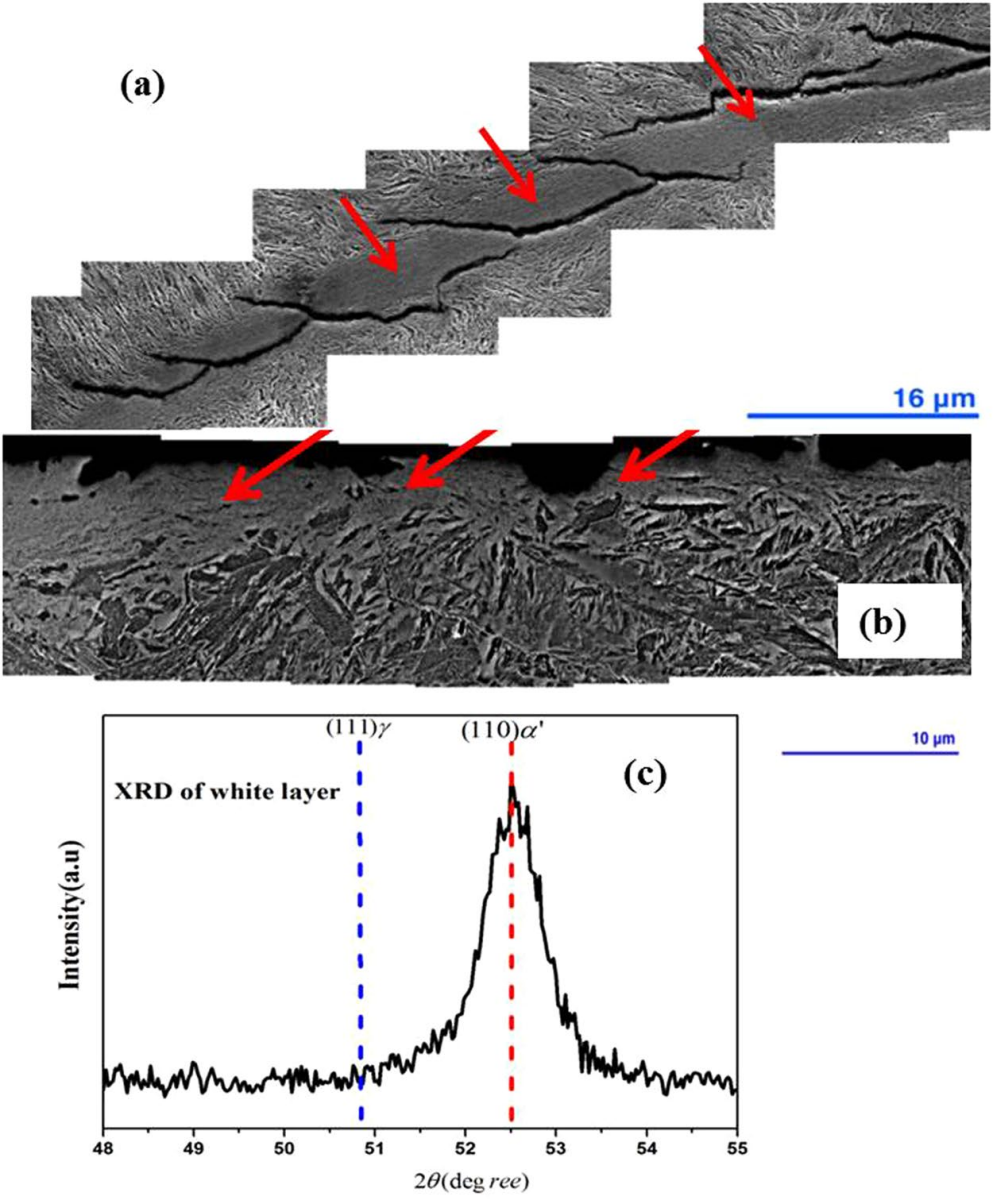
SEM images of the deformed sample featuring (**a**) the crack only propagates through the white layer and is stopped by the base material, (**b**) There is an abrupt transition from the base material to the white layer from where the material chipped off. (**c**) XRD diffraction pattern for the white layer featuring a martensite peak only. Red arrow indicates the white layer in (**a**) and (**b**).

Figure 6a shows that cracks propagate within the white layer and along the interface between the white layer and the base material. Crack propagation within the white layer seems to be terminated on contact with the base material, or alternatively cracks propagate along the interface. No flow lines are visible in Fig. 6b, but there is an abrupt microstructural transition from the base material to the white layer. It is likely that in this instance, a crack formed in the white layer and that it has chipped off (spalled) from the base material.

Figure 6c shows the XRD pattern of a thin layer cut from the highly deformed area and the presence of a martensite peak only, suggests the retained austenite (see Fig. 3) has been completely transformed to martensite. In order to fully understand the mechanism of the severely deformed structure and the consequent structural failure, it is critical to identify the microstructural changes occurring within and in the vicinity of the cracks and to determine their relationship with the deformed base material.

### EBSD of the white layer

The grains in the vicinity of the cracks are typically a few nanometers to several hundred nanometers in width. An EBSD map of the impacted samples depicts the microstructural changes accompanying strain localization and the formation of recrystallized grains and cracks in the steel under dynamic impact loading. The corresponding strain rates generated in the specimens by these impact loads suggest variations in the mechanical response across the sample, which can be estimated using microstructural analysis of the areas close to, and remote from the crack.

**Figure 7:**
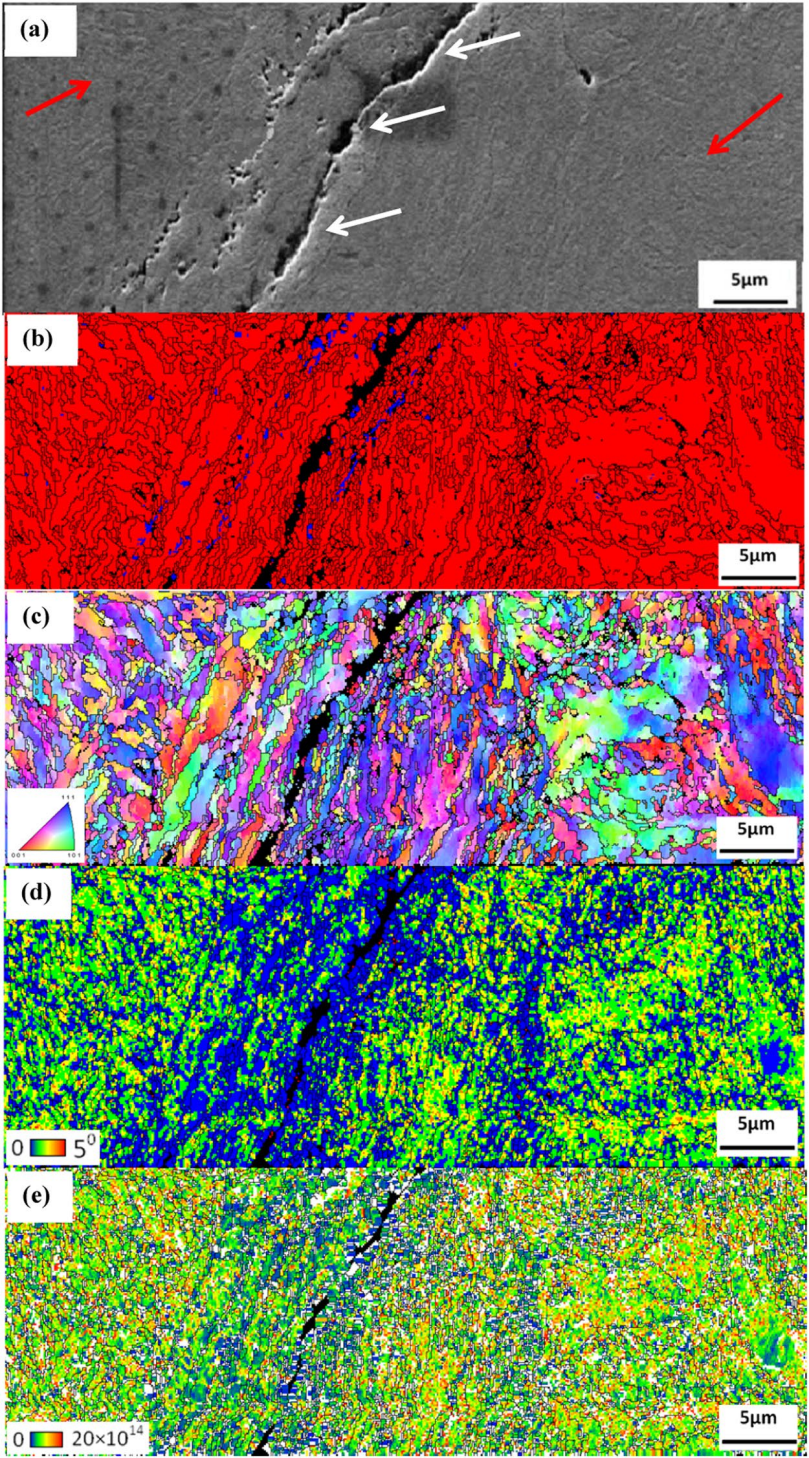
EBSD patterns of the steel following severe plastic deformation (**a**) Secondary electron image of cracks that formed in the vicinity of the white layer. White arrows indicate the cracked region and red arrows indicate the base material. (**b**) Phase map. Different phase has different colours (blue- austenite; red- martensite) (**c**) Inverse pole figure (IPF) (**d**) KAM map and (**e**) Dislocation density map. Black lines are grain boundaries with a misorientation angle of more than 15°.

A secondary electron image of cracks that formed in the vicinity of the white layer is shown in Fig. 7(a). The white arrows point to the position of the white layer and red arrows indicate the area some distance away from the highly deformed area (outside white layer). The phase map in Fig. 7(b) reveals that the structure consists mostly of martensite. Hence, almost complete transformation of austenite to martensite occurred in the material immediately adjacent to the crack in the white layer, while a thin layer of nano-sized austenite was identified between the white layer and the base material. By this HR-EBSD study, we identified for the first time, a hybrid structure of the white layer, in which nano sized martensite and retained austenite coexist.

The IPF map, Fig. 7(c) of the martensite phase inside and outside of the white layer shows grains of random orientation and there is no evidence of preferred orientations.

Figure 7(d) shows that the KAM map and Fig. 7(e) is the dislocation density map in the material outside the white layer is higher than $20\times {10}^{14}{m}^{-2}$, high enough to argue that slip is the dominant mechanism of plastic deformation mechanism in the high carbon steel samples investigated in this study. It is worth noting that the morphology of the martensite inside and close to the white layer is not lath or plate like martensite such as typically observed in undeformed martensite. Grains with a near to zero dislocation density (blue colour) provided evidence of recrystallized grains in the impacted specimen. There is a distinct change in martensite morphology from base material on approach to the white layer. This observation provides evidence that the original retained austenite transforms to martensite and is then elongated as a result of severe plastic deformation. No recrystallized grains were observed at larger distances from the white layer and the average grain size in these areas is almost the same as that of the undeformed grains.

Although the phase maps revealed that the phase transformation of retained austenite to martensite occurred within and outside the white layer region in all specimens, a thin layer of nano grain sized austenite is visible in the transition region between the shear band and the base material. This observation is very important since it provides evidence that new austenite grains might have formed on heating the martensitic structure into the austenite phase field. It is conceivable that the heat generated by severe plastic deformation results in localized areas reaching the phase transition temperature and that new austenite grains can form indeed. Further evidence that the severe plastic deformation resulted in a significant increase in temperature is to be found in the observation that certain martensitic regions have been observed that contained very low dislocation densities, pointing to such an increase in temperature that partial recrystallization of the martensite occurred, in line with earlier suggestions^22^.

### TKD and TEM of the white layer and dislocation structure

Transmission Kikuchi diffraction enabled automated orientation analyses of nanostructured materials to be conducted in the SEM on a TEM sample^22^. The thickness of the TEM sample plays a vital role in determining the quality of the diffraction patterns and it is therefore important to note that these TKD scans were conducted on the TEM foil with a sample thickness of ~70nm. The sample was cut from the white layer shown in the SEM images. The step size chosen for the scanning was ~5nm. These refined and high resolutions TKD scans made it possible to detect the grain structure and morphology of the white layer.

**Figure 8:**
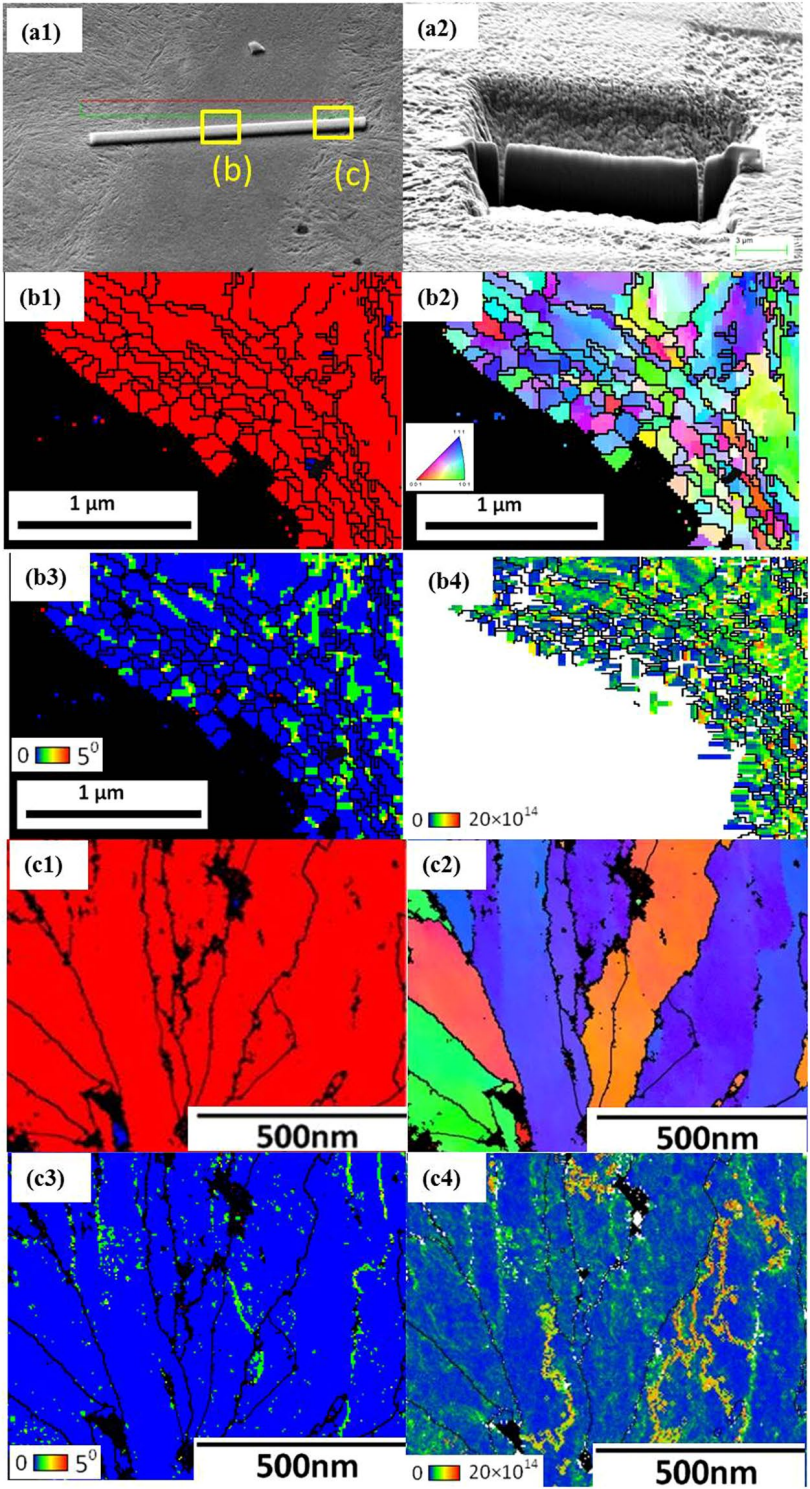
SEM image and EBSD patterns of severely deformed sample with white layer (marked with (**b**) yellow square) and adjacent area (marked with (**c**) yellow square) which has been obtained by TKD investigation (a1) showing the position of the foil relative to the white layer and the positioning and the platinum deposition (a2) The TEM foil prepared by FIB techniques from the region that contains the white layer (b1) EBSD phase map for the white layer area (b2) Inverse pole figure (IPF) for the white layer area (b3) KAM map for the white layer area and (b4) Dislocation density map for the white layer area. (c1) EBSD phase map for the adjacent area (c2) Inverse pole figure (IPF) for the adjacent area (c3) KAM map for the adjacent area and (c4) Dislocation density map for the adjacent area. The black lines are grain boundaries with a misorientation angle of more than 15°.

Figure 8 shows the TKD (Transmission Kikuchi diffraction) images of the area within the white layer (taken at position b which has been markes with yellow square with b in Fig. 8(a1) and all the (b) figures are for this reagion), and in the adjacent region of white layer (taken at position c which has been markes with yellow square with c in Fig. 8(a1) and all the 8(c) figures are for this reagion). Figure 8(b)s and (c)s reveal two distinctly different morphologies of the microstructure. However, in both cases severe plastic deformation has caused the retained austenite to transform to martensite.

The grains within the white layer, shown in Fig. 8b1 and Fig. 8(b2), are much smaller than those in the lightly strained area of the base metal outside the white later shown in the EBSD micrograph (Fig. 7, indicated by the red arrows). The very small equiaxed grains (~50 nm) shown in Fig. (b1) have misorientations of more than 15° with neighbouring grains, and hence, they have negligible dislocation density.

Conversely, the morphology and structure of the elongated grains in the region adjacent to the white layer shown in Fig. 8(c1) and (c2), as are quite different. This structure provides evidence highly strain-induced elongation with typical grain dimensions of about 50nm to 100nm in width. The grain and sub-grain structures are clearly distinguishable in the IPF colour mappings. Figure 8(c2) implies that the dislocation density accumulated continuously as a result of the severe deformation within the sub boundaries due to an increase in misorientation.

**Figure 9:**
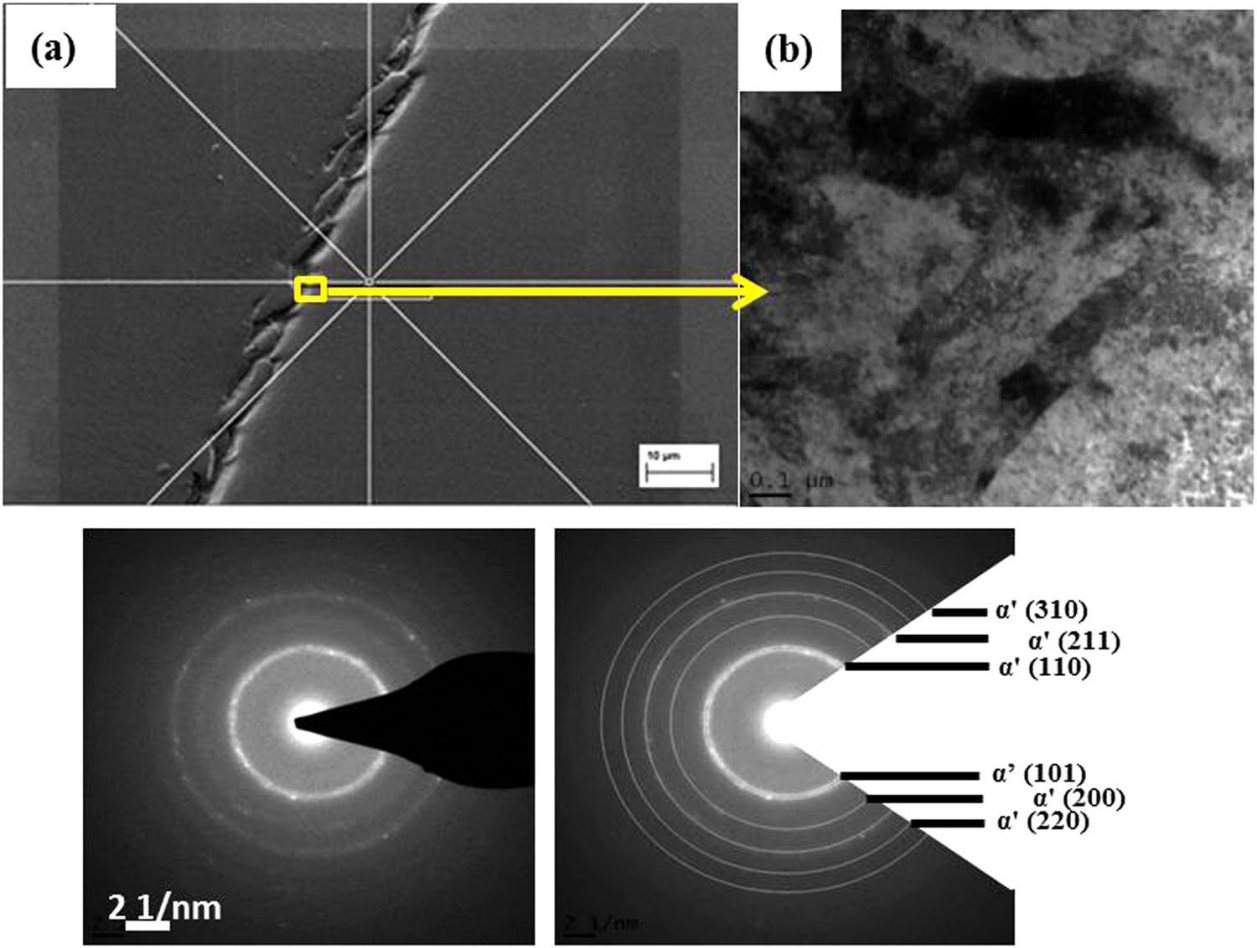
(**a**) The position for TEM foil prepared from the region that has white layer using FIB. (**b**) Bright-field TEM image of the white layer (**c**) Diffraction pattern of the white layer and (**d**) indexing of the diffraction pattern.

In some places the grains of white layer are only a few nanometres in width and in order to investigate the structural evolution of the white layer in more detail, TKD analyses were conducted. A TEM sample was prepared from the cracked surface where the flawless white layer could be identified. Figure 9 shows the TEM images and diffraction pattern of the nanostructure within the white layer. The selected area diffraction (SAD) pattern in Fig. 9(d) shows a ring pattern of the α’ (BCT martensite) phase with some brighter dots along the circumference, indicating some preferred orientation. No evidence was found of the presence of the γ-austenite phase.

**Figure 10:**
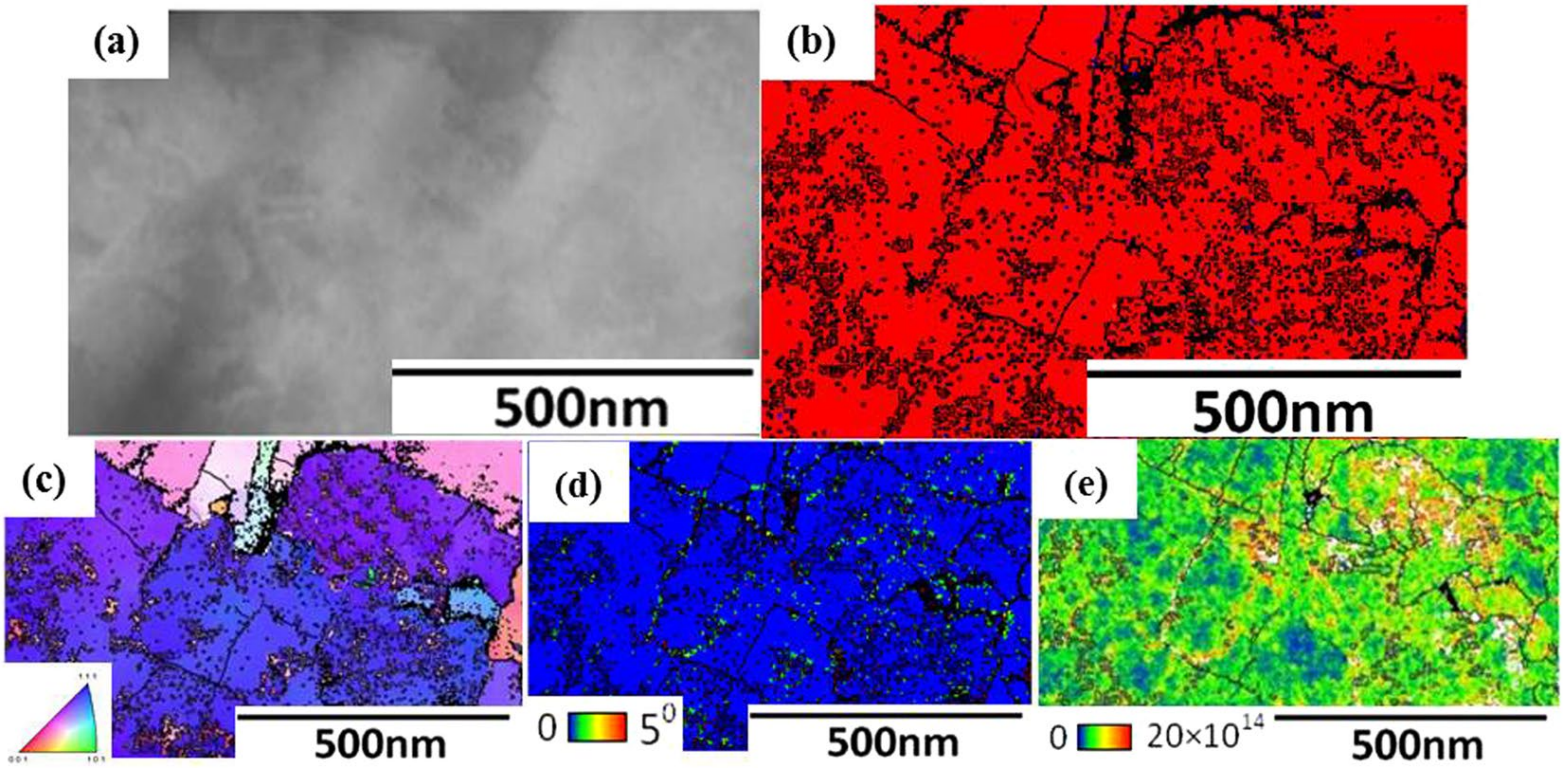
(**a**) SEM image of the white layer (**b**) EBSD phase map of white layer which has been obtained by TKD investigation, different phases are identified by different colours (blue- retained austenite; red- martensite) (**c**) Inverse pole figure, (**d**) KAM map and (**e**) dislocation density within grains in the white layer. Black lines are grain boundaries with a misorientation angle of more than 15°.

Further TEM/TKD investigations revealed that there are variations in the texture and morphology of the white layer from cracked zone. Figure 10 reveals the presence of grains with dimensions similar to those shown in Fig. 8. However, some ultra-fine grains, less than 20nm in diameter were found within these larger grains as shown in Fig. 10(c),(d),(e). Figure 10(e) reveals that these nano-sized crystallites have a dislocation density as high as $20\times {10}^{14}{m}^{-2}$.

**Figure 11:**
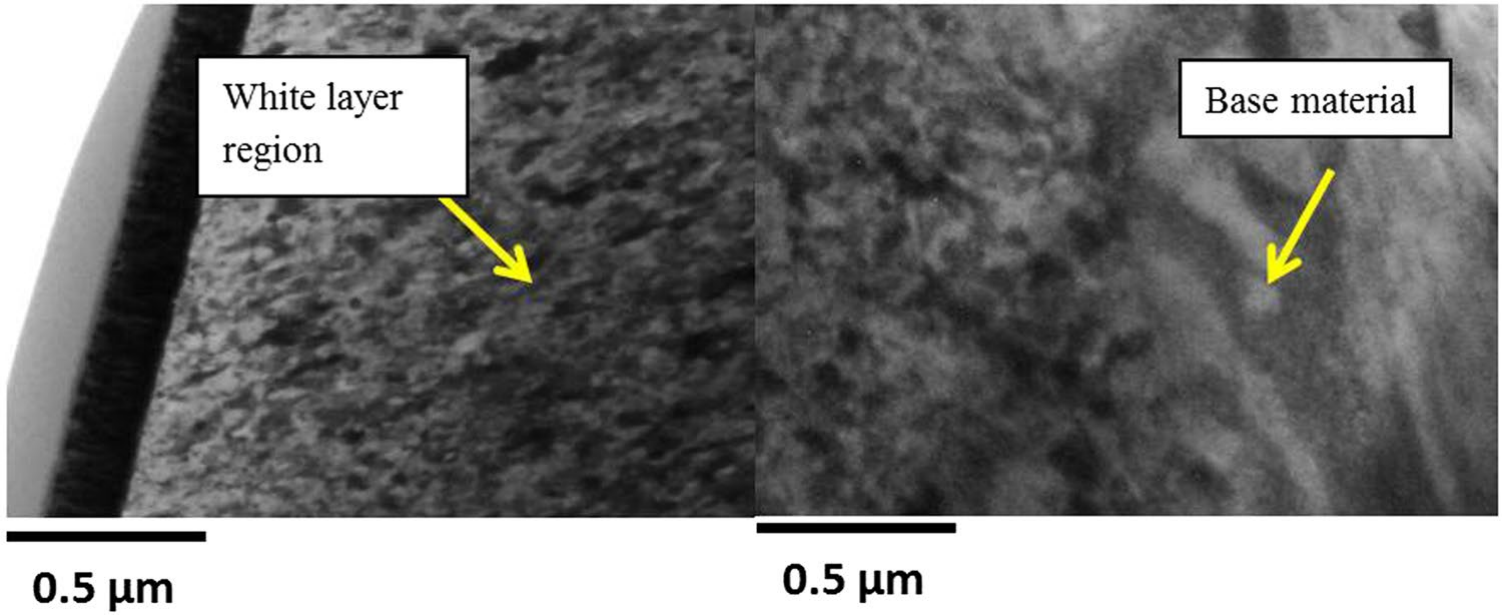
Bright field TEM image showing white layer and base material region.

We also collected TEM sample of the white layer from the uncracked zone to confirm the nano structure within the white layer as it has shown in Fig. 11. The bright field image shows the significant structural refinement in the white layer where the grains are in nano-meter size.

**Figure 12:**
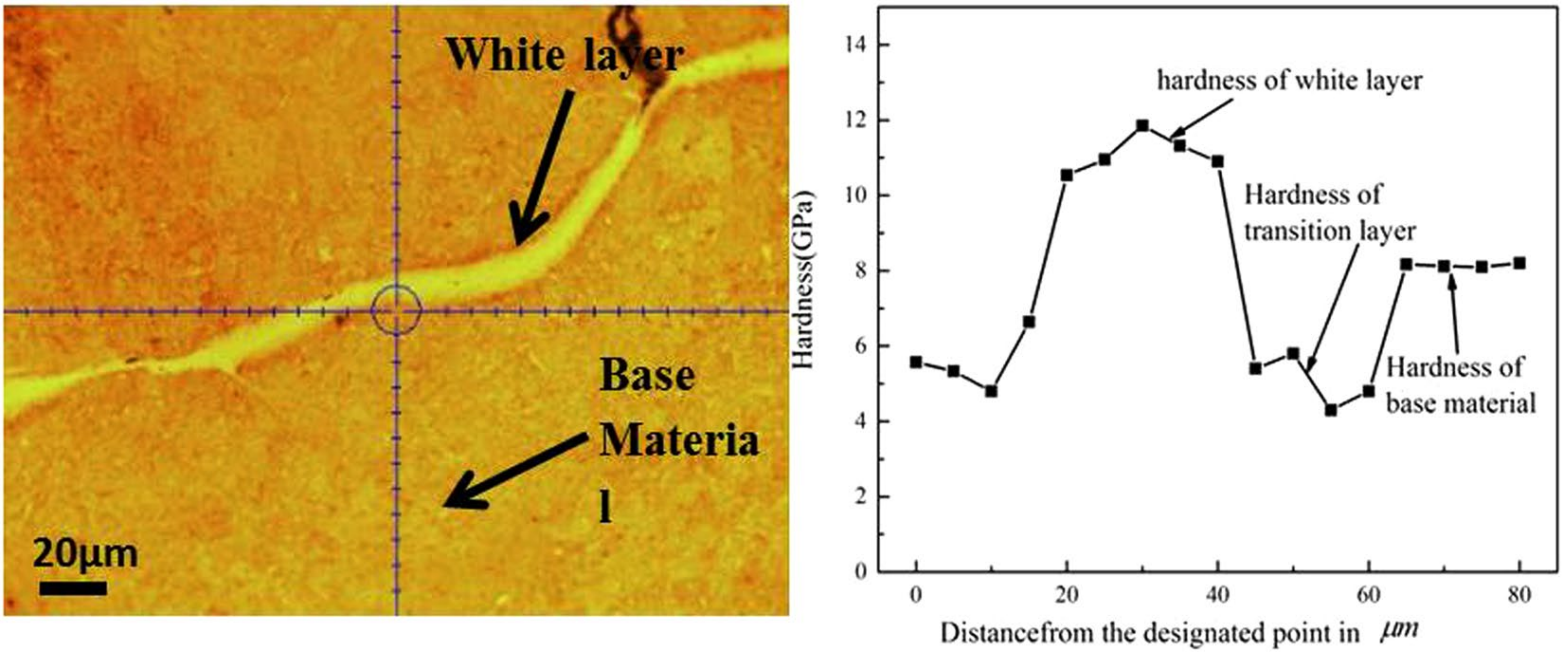
Nano hardness profile of white layer and the adjacent area.

In order to better understand the deformation mechanisms leading to the formation of the nanostructured white layer, an attempt was made to determine the mechanical properties of the white layer and the regions in the immediate vicinity of this layer. To this end, a nano-indentation technique was used to determine the hardness of the white layer and that of the adjacent areas. Nano-indentations were made as per the array of indents shown in Fig. 12.

The hardness of the material adjacent to the white layer varied between 8.06GPa and 8.12GPa whereas the hardness of the white layer region varied from 10.5GPa to 12GPa (i.e. some 20-40% higher).

Figure 12 shows that there is a softer region immediate adjacent to the white layer. It was shown in the EBDS image of Fig. 4 that there is a thin layer of austenite adjacent to the white layer. This austenitic region is marked with a green arrow in Fig. 4(a) and the lower hardness of the area adjacent to the white layer is most likely due to the presence of this austenite layer. However, heat generated during severe plastic deformation, might also lead to tempering of the martensite adjacent to the white layer as was suggested by Li *et al* ^23^.

### Formation mechanism of nano-crystalline grains

Metallographic observations of the white layer and surrounding areas have shown that extensive plastic flow occurred in the direction of impact and that the resulting nano-crystalline structure is primarily mechanically induced. The severely deformed microstructural development outlined by the experimental observations above can be accounted for as follows:

The microstructure of the undeformed, high carbon steel under investigation consists of martensite and a large fraction of retained austenite (Fig. 3). During repetitive and severe impact loading, significant plastic flow occurs as shown in Fig. 4(b). The retained austenite is transformed to martensite by this high energy impact, with a concomitant refinement of the martensitic structure (Fig. 4). Further severe plastic deformation seems to produce a white layer with an ultra-fine grain structure. This white layer is brittle and susceptible to cracking as shown in Fig. 6. At first sight it appears that the white layer is solely mechanically induced. However, we have found convincing evidence of significant heat generation in the area within and surrounding this white layer: Fig. 7(b) shows that some austenite grains have been formed following severe impact of an originally martensitic structure. This observation indicates that new austenite grains could have formed during heating as a result of the severe impact energy imposed in the sample. Further evidence of the premise that the area in and in close proximity to the white layer has been heated to relatively high temperatures, is to be found in Fig. 8(b) where it is clear that very fine, equiaxed grains with very low dislocation densities are present. This observation strongly suggests that these small, equiaxed grains are the result of the initial stages of recrystallization. Kikuchi diffraction analyses revealed the presence of extremely small grains within the white layer (Figs 10 and 11) providing further evidence that the white layer comprise nano-sized crystallites. The white layer is significantly harder that the base material as shown in Fig. 12 and moreover, the area in the immediate vicinity of the white layer is not only significantly softer than the white layer itself, but it is also softer that the matrix, an observation that provides more experimental evidence that significant heating has occurred. It is worth noting that it in this very area that small austenite grains as well as small (almost) dislocation free nano-sized crystallites have been observed. The intensity of the impact energy clearly determines the thickness of the white layer, which can be created by a single or alternatively multiple high-energy impacts, depending on the impact force and angle. These different impacting events lead to inhomogeneous distributions of stress and heat generation and are most probably the underpinning reason for the formation of the largely inhomogeneous white layer structure.

## Conclusion

- This study has shown that a white layer can form as a result of severe plastic deformation in a dynamic impact test.
- Significant plastic flow leads to a severely deformed, nano-crystalline structure.
- Convincing experimental evidence was found that there is a significant increase in the temperature in and in the immediate vicinity of the white layer.
- Nano-crystallites are formed either by heating into the austenite phase-field, thereby yielding very fine grains, or alternatively by early recrystallization events, again leading to nano-sized grains.
